# Concordance of skin test reactivity between indoor inhalant allergens among children with allergic respiratory disease

**DOI:** 10.1186/s12887-021-02800-2

**Published:** 2021-08-11

**Authors:** Prapasri Kulalert, Paskorn Sritipsukho, Sira Nanthapisal, Orapan Poachanukoon

**Affiliations:** 1 Department of Clinical Epidemiology, Faculty of Medicine, Thammasat University, Pathumthani, 12120 Thailand; 2 Division of Allergy and Immunology, Department of Pediatrics, Faculty of Medicine, Thammasat University, Pathumthani, Thailand; 3 Center of Excellence in Applied Epidemiology, Thammasat University, Pathumthani, Thailand; 4 Center of Excellence for Allergy, Asthma and Pulmonary Disease, Thammasat University, Pathumthani, Thailand

**Keywords:** Asthma, Allergic Rhinitis, Allergens, Skin Tests

## Abstract

**Background:**

In vitro studies have demonstrated cross-reactivity among indoor allergen proteins in children with allergic respiratory diseases. However, there are only few studies evaluating in *vivo* response. A skin prick test (SPT) with commercial indoor solutions is widely used in clinical practice. We aimed to evaluate SPT agreement in children with allergic respiratory disease between pairs of common indoor allergens.

**Methods:**

We reviewed SPT results of children 2 to 18 years old, diagnosed with respiratory allergic disease. Results from house dust mite *(Dermatophagoides farinae, Dermatophagoides pteronyssinus),* cockroach (*Periplaneta americana*, *Blatella germanica*), cat and dog were collected. Sensitization was defined as ≥ 3 mm in wheal diameter. Kappa coefficient (κ) was used to analyze sensitization concordance for each allergen pair.

**Results:**

The charts of 300 children, 187 (62.33%) males, were reviewed. Mean age was 7.43 ± 3.29 years with 183 (61%), 140 (46.67%), 45 (15%), 30 (10%) sensitizations to house dust mite (HDM), cockroach, cat and dog, respectively. Sensitization concordance between HDM and cockroach was moderate: κ = 0.53 (95% CI: 0.42–0.64). Moderate agreement occurred between dog and cat: κ = 0.41 (95%CI: 0.30–0.52). HDM-sensitized children showed poor concordance with both cat κ = 0.17 (95%CI: 0.09–0.24) and dog κ = 0.09 (95%CI: 0.03–0.14). There was also poor concordance between cockroach-sensitized children to cat κ = 0.19 (95%CI; 0.11–0.28) and dog κ = 0.11 (95%CI; 0.04–0.18).

**Conclusion:**

We demonstrated moderate agreement of SPT response between HDM and cockroach as well as dog and cat. This may be due to cross-reactivity. Component-resolved diagnosis should be considered in children with co-sensitization of these allergen pairs.

## Background

Asthma and allergic rhinitis are common allergic respiratory diseases in children. Exposure to aeroallergens especially indoor allergens are closely linked to sensitization, a significant risk factor for the triggering and persistence of symptoms associated with allergic respiratory diseases [1–3].

Indoor allergens are derived from house dust mite (HDM), cockroach and domestic pets. HDM are the most prevalent cause of sensitization worldwide, especially *Dermatophagoides farina*e and *Dermatophagoides pteronyssinus*. Cockroach sensitization is second most frequent for children with allergic respiratory diseases, which include American cockroach (*Periplaneta americana*) and German cockroach (*Blattella germanica*). Cat and dog present the most notable child pet allergies [3, 4]. In vitro studies have demonstrated cross-reactivity in protein families between arthropod inhalant allergens *i.e.* HDM and cockroach or between mammalian allergens *i.e.* cat and dog [4–7].

A skin prick test (SPT) is recommended to diagnose allergen sensitization. It is widely used in clinical practice because of its many advantages: easy to perform, fast results, and low cost [8]. Extracts from *Dermatophagoides farina*e, *Dermatophagoides pteronyssinus Periplaneta americana*, *Blattella germanica*, cat and dog are readily available.

However, there has been few studies evaluating the correlation of skin test responses among indoor allergens. In our opinion, it would be useful to obtain more comprehensive data evaluating indoor allergen SPT results among children with allergic respiratory diseases. Our hypothesis is SPT response concordance may be associated with cross-reactivity among protein family allergens. The objective of this study was to evaluate the concordance of skin test response between the pairs of indoor allergens among children with allergic respiratory diseases.

## Methods

This was a retrospective medical chart review of children, 2 to 18 years old, diagnosed with allergic rhinitis and/or asthma having undergone SPT at the Pediatric Allergy Clinic of Thammasat University Hospital, Pathum Thani Province, Thailand, from January 2015 to January 2017.

### Data collection

Data on patient age when performed SPT, gender and diagnosis were collected. SPT results for 6 extracts, include, *Dermatophagoides farinae* 10,000 AU/mL*, Dermatophagoides pteronyssinus* 10,000 AU/mL*,* American cockroach 1:20 w/v*,* German cockroach 1:20 w/*v,* Dog epithelium 1:20 w/v, and Standardized cat hair 10,000 BAU/mL*.* Histamine 1 mg/mL and 50% Glycerine were used as positive and negative control. All SPT solutions were obtained from ALK-Abelló, Port Washington, New York, USA. All patients had SPT on the volar of the forearm using lancets by a team of experienced nurses with each patient having the same nurse and all extracts in the same time. Readings were performed 15–20 min after SPT. Wheal size was measured by the longest and orthogonal diameters, reported as millimeters (mm).

### Definition of outcome

Sensitization was defined as when the wheal diameter was ≥ 3 mm than that of the negative control. HDM sensitization was defined as a positive to SPT result for *Dermatophagoides farinae* and/or *Dermatophagoides pteronyssinus.* Cockroach sensitization was defined as positive SPT result for American cockroach and/or German cockroach*.*

### Statistical analysis

Demographic characteristics were presented as mean ± SD for continuous variables and as % for categorical variables. Prevalence of sensitization was reported as frequency (%). Sensitization agreement for each allergen pair used kappa coefficient (κ) for analysis: κ < 0.00 was considered poor strength of agreement; κ: 0.00 – 0.20 slight strength; κ: 0.21 – 0.40 fair; κ: 0.41 – 0.60 moderate; κ: 0.61 – 0.80 substantial; and κ: 0.81 – 1.00 almost perfect agreement [9].

### Sample size calculation

Sample size was estimated using power analysis for a one sample proportion test in Stata v15.1. We hypothesized moderate agreement for skin test response between pairs of cross-reactivity protein family allergens. Thus, κ was estimated 0.5 with probably not expected to have deviated more than 0.1. The number of population that must be used at least was 259 participants for providing the 90% power, α = 0.05 and two-side test.

## Results

The medical charts of 300 patients were reviewed. Mean age was 7.43 ± 3.29 years; 187 (62.33%) were males, and 113 were females (37.67%). Two hundred and sixteen (72.0%) patients were diagnosed with allergic rhinitis alone, and 13 patients (4.33%) were diagnosed with asthma alone. Seventy-one patients (23.67%) had both allergic rhinitis and asthma. In all, 183 (61.00%) patients were sensitized to HDM, 140 (46.67%) were sensitized to cockroach, 45 (15.00%) were sensitized to cat hair, and 30 (10.00%) were sensitized to dog epithelium (Table 1).

**Table 1:** Patient demographics (N = 300)

Characteristics	N (%)
Age (mean ± SD in years)	7.43 ± 3.29
Gender	
Male	187 (62.33)
Female	113 (37.67)
Diagnosis	
Allergic rhinitis only	216 (72.00)
Allergic rhinitis with asthma	71 (23.67)
Asthma only	13 (4.33)
Skin test sensitization	
House dust mite	183 (61.00)
Cockroach	140 (46.67)
Cat hair	45 (15.00)
Dog epithelium	30 (10.00)

### Co-sensitization and concordance of SPT reactivity to indoor allergens

Results showed in Tables 2 and 3, which describe the concordance of SPT responses between each allergen pair. There were 126/183 (68.85%) patients who were sensitized to HDM having co-sensitization with cockroach. One hundred and twenty-six (126) HDM-sensitized patients had cockroach sensitization: co-sensitization was 90%. SPT responses between HDM and cockroach had a moderate agreement, κ = 0.53 (95%CI: 0.42 to 0.64).

**Table 2:** Indoor allergen co-sensitization frequencies

Patient sensitization	Number of patients with co-sensitization, N (%)			
	**House dust mite**	**Cockroach**	**Cat hair**	**Dog epithelium**
**House dust mite (N = 183)**	-	126 (68.85)	42 (22.95)	26 (14.21)
**Cockroach (N = 140)**	126 (90.00)	-	35 (25.00)	22 (15.71)
**Cat hair (N = 45)**	42 (93.33)	35 (77.78)	-	18 (40.00)
**Dog epithelium (N = 30)**	26 (86.67)	22 (73.33)	18 (60.00)	-

**Table 3:** Skin test responses agreements between indoor allergen pairs

**Allergen**	**Kappa (95% CI)**			
	**House dust mite**	**Cockroach**	**Cat hair**	**Dog epithelium**
**House dust mite**	-	0.53 (0.42 to 0.64)	0.17 (0.09 to 0.24)	0.09 (0.03 to 0.14)
**Cockroach**	-	-	0.19 (0.11 to 0.28)	0.11 (0.04 to 0.18)
**Cat hair**	-	-	-	0.41 (0.30 to 0.52)

In contrast, 22.95% (42/183 patients) of those with sensitization to HDM also had sensitization to cat hair, while 14.21% (26/183) had sensitization to dog epithelium. Thus, concordance of SPT responses between HDM and all pet allergens tested were poor: κ = 0.17 (95%CI: 0.09 to 0.24) for cat hair and 0.09 (95%CI; 0.03 to 0.14) for dog epithelium. Only 25% (35/140 patients) of those with cockroach sensitization had cat hair sensitization, and 15.71% (22/140) were sensitized to dog dander. This also showed a poor agreement in SPT responses between cockroach and cat hair: κ = 0.19 (95% CI: 0.11 to 0.28) as well as between cockroach and dog epithelium: κ = 0.11 (95%CI: 0.04 to 0.18).

Among patients sensitized to pet allergens, 40% of the children (18/45) with cat hair sensitization showed coexistence with dog epithelium sensitization. Around 60% (18/30) with dog epithelium sensitization showed cat hair sensitization, too. Concordance of SPT responses between cat and dog were moderate: κ = 0.41 (95% CI: 0.30 to 0.52).

## Discussion

Our study showed HDM to be the most common indoor allergen sensitization, followed by cockroach, cat and dog. This pattern of indoor allergen sensitization has not appeared to change over time as our results are similar to previous studies for children living in Thailand [10, 11] and other Asian countries [3].

*Dermatophagoides farinae* (Der f) and *Dermatophagoides pteronyssinus* (Der p) were common sensitization in Thai atopic patients [11, 12] and were the most identified from house dust samples [13]. *Blomia tropicalis* (*Blo t*) predominantly found in tropical and subtropical regions [14]. However, a previous study indicated that *Blo t* was rarely found in Thailand [13]. Previous study reported low prevalence of *Blo t* sensitization and all patients with sensitized to *Blomia troplicalis* extracts were sensitized to *Dermatophagoides* [12].

Cockroach is the second most common aeroallergen sensitization. Our study was evident in 46.67%. The prevalence of cockroach sensitization varies in different countries.

The high sensitization rate was found in Brazil 57.5% [15], Africa (55%) [16], Taiwan (50.7%) [17], and US (42%) [18]. In contrast, this prevalence was higher than that found in other Asian countries *e.g*. Hong Kong (33%) [19], China (24.3%) [20], Korea (23.6%) [21], India (18.3%) [22], and Vietnam (13.1%) [23], respectively. Our sensitization rate was also higher than European countries *e.g*. Poland (25%) [24] and Spain (15%) [25].

Cockroach exposure has been linked to cockroach sensitization [26]. The levels of cockroach allergens measure in the home are strongly associated with a greater risk for the development of cockroach sensitization [27, 28]. In Thailand, Tungtrongchitr A et al. found that cockroach allergens of the predominant species, *Periplaneta Americana*, were detected in all households of allergic patients, with the highest level in the kitchen areas. The mean allergen level in kitchen dust were 62.8 µg per g of dust [29]. This may potential for development of cockroach sensitization.

We had a high proportion of co-sensitization between HDM and cockroach. This is also similar to prior researches. Uzel A, et al. [30] reported 73.9% of adults with cockroach sensitivity had reactivity to HDM. An evaluation by Macan J, et al. stated positive SPT to HDM denoted significantly increased risks for reactivity to cockroach [31]. Moreover, a large cohort study (N = 5,782) confirmed the association between sensitization to cockroach and mite was strong, 71% of those with a positive SPT to cockroach also had a positive SPT to any mite. Conversely, of mite-sensitized subjects 36% were also sensitized to cockroach [23]. However, these previous studies did not evaluate the level of agreement in pair allergens *i.e.* SPT responses between HDM and cockroach allergens. Our study is the first publication to do so, demonstrating moderate agreement.

The rationale of agreement for skin test reactivity between HDM and cockroach could be explained by protein family cross-reactivity sharing epitopes *e.g.* tropomyosin [32]. Studies reported Group 10 allergenic tropomyosin found in HDM [4, 5], namely, *dermatophagoides farinae* (Der f 10) and *dermatophagoides pteronyssinus* (Der p 10). Cockroach found allergenic tropomyosin in *periplaneta americana* (Per a7), *blattella germanica* (Blag7) [6].

Sun BQ et al. [33] reported 88% of positive SPT to cockroach patients were also positive SPT to HDM. An IgE cross-inhibition study confirmed that Der p sensitization may cause false positive SPT reactions against cockroach.

Allergen extracts from HDMs are frequently of poor quality. The use of purified recombinant allergens for diagnostic purposes may therefore be considered as an alternative, or even an improvement over the traditional allergen extracts [34] Weghofer et al. reported 10–18% of mite allergic patients in Europe had IgE-reactivity to Der p 10 (mite tropomyosin). Westritschnig et al. [35] demonstrated that 55% of African patients had Der p 10 (mite tropomyosin) sensitization which are higher than European study.

Diagnosis of cockroach allergy is performed using crude extracts by in vivo skin testing and/or in vitro measurement of specific IgE to cockroach (by ImmunoCAP). Cockroach extracts are non-standardized, highly variable in allergen content and show low potency. Recombinant cockroach allergens have been successfully used for assessment of sensitization [36]. The high frequency of reactivity to cockroach tropomyosin seen in Brazil could reflect cross-reactivity to mite tropomyosin, which shares 80% sequence identity to the cockroach homolog [37].

In addition, tropomyosin represents a cross-reactive allergen also found in crustaceans (e.g. shrimp, lobster, crab), and helminths [38–41]. However, we did not evaluate the association between respiratory indoor allergens with crustaceans and helminths. Further research is needed.

Cats and dogs are the most prevalent household pets [42]. Pet ownership and animal allergen exposure was associated with corresponding allergic sensitization [43–45]. The prevalence of sensitization to cats and dogs of our study were low (15% for cat and 10% for dog). Our results are consistent with a previous study [11]. Sritipsukho et al. reported the low prevalence of pet sensitization (13% for cat and 8% for dog) and low prevalence (20%) of pet ownership in our country. We assumed the low prevalence of pet ownership may be causing of the low sensitization to pets allergen.

We noted moderate SPT concordance between cat and dog. The co-sensitization to dog was 60% among patients sensitized to cat. Sixty-eight of 109 patients (62%) with animal allergy showed IgE reactivity to cat allergens and dog allergens [46]. To date, molecular diagnosis is strongly recommended performing in polysensitized patients to distinguishing between sensitizations specific to singular species and sensitizations due to cross-reactivity [47]. The frequency of co-sensitization with cat and dog may be explained by shared proteins between the two species e.g. lipocalins, or serum albumins. Four dog allergens (e.g. Can f 1, Can f 2, Can f 4, and Can f 6) and two cat allergens (Fel d 4 and Fel d 7) are in the lipocalins family of proteins.

Studies have shown lipocalins are responsible for allergenic protein cross-reactivity between cat and dog dander. Smith W et al. found that Fel d 7 binds IgE in 38% of cat allergic individuals. Fel d 7 share 62% sequence identity with Can f 1 and may suggest a molecular mechanism of cross-reactivity and cosensitization [48].

Can f 6 showed cross-reactivity with Fel d4. sIgE to Can f 6 is present in 38% of patients sensitized to dogs; however, it appears in 60% of patients sensitized to both cats and dogs, which could be related to its identity with Fel d 4 [49]. In addition, albumins are minor allergens. Allergenic serum albumins also include Can f3 (Dog) and Fel d2 (Cat) [50].

Moreover, Cat and dog may be sensitized to other animals e.g. horse. Some lipocalins have amino acid sequences with up to 60% identity, which explains the cross-reactivity between them, for example, Can f 6 (dog), Equ c 1 (horse), Fel d 4 (cat), Ory c 4 (rabbit), Mus m 1 (mouse), Rat n 1 (rat) [51]. The further study should be explore the association of these mamal animals.

Apart from that, we must point out causing co-sensitization as well as from co-exposure. Studies detected multiple indoor allergens, *e.g.* Der f1, Der p1, Bla g1, Can f1, Fel d1, etc., form dust sample of the participant’s home [52, 53]. Co-sensitization among indoor allergens is related to the exposure of multiple indoor allergens in house environment. Therefore, concordance of SPT results of indoor allergens may be due to their cross-relativities.

Identification of allergen sensitization remains important for education on allergen avoidance and must be considered in specific allergen immunotherapy. In routine practice, SPT with indoor allergen extracts is the primary tool for detecting sensitization, and allergen avoidance for all sensitized allergens is always recommended. We demonstrate clear SPT response agreement between certain indoor allergens with shared protein families.

Recently, Component-resolved diagnosis (CRD) has been emerged, which is based on the determination of serum IgE concentration against individual components of the allergen. CRD is potential for distinguishing true allergens from cross-reactive allergen molecules [54]. Several studies have shown the usefulness of CRD in allergy to furry animals, mites and arthropods [55–57].

Best on our knowledge, our study is the first publication showed in vivo evidence of the agreement between the pairs of indoor allergens among children with allergic respiratory diseases. These implications emphasize physicians should consider more specific testing in those with co-sensitization between HDM and cockroach as well as cat and dog dander; this would render a more accurate diagnosis and exclude possible cross-reactivity. The limitation of our study, we should note our researchers reviewed retrospective charts lacking clinical severity data when SPT were performed, making them unable to evaluate the exact relationship between co-sensitization concordance and disease severity or the intensity of the wheal diameter with clinical relevance. As such, it would helpful for further studies to be prospective.

## Conclusion

Our study showed moderate concordance of SPT between HDM and cockroach as well as between dog and cat, most likely due to cross-reactivity, or possibly parallel sensitization. CRD should be considered in children with co-sensitization of these allergen pairs.

## Data Availability

The dataset analyzed in the current study is available from the corresponding author on reasonable request.

## Abbreviations

SPT: Skin prick test
CRD: Component-resolved diagnosis
HDM: House Dust Mite
